# Role of neutrophils in regulating vascular permeability in inflammatory and autoimmune diseases

**DOI:** 10.1007/s00011-025-02157-7

**Published:** 2026-02-07

**Authors:** Reza Akbarzadeh, Manu Beerens, Jens Y. Humrich, Kristina Kusche, Peter Lamprecht, Gabriela Riemekasten, Thomas Renné

**Affiliations:** 1 Department of Rheumatology and Clinical Immunology, University of Lübeck, Lübeck, Germany; 2 Institute of Clinical Chemistry and Laboratory Medicine, University Medical Center Hamburg-Eppendorf, Hamburg, Germany; 3 German Research Centre for Cardiovascular Research (DZHK), Partner Site Hamburg/Lübeck/Kiel, Lübeck, Germany; 4 Institute of Physiology, University of Lübeck, Lübeck, Germany; 5 Irish Centre for Vascular Biology, School of Pharmacy and Biomolecular Sciences, Royal College of Surgeons in Ireland, Dublin, Ireland

**Keywords:** Neutrophil, Vascular permeability, Inflammation, Autoimmune diseases

## Abstract

**Background:**

Regulation of vascular endothelial permeability is crucial for maintaining hemostasis and controlling extravasation of immune cells in response to injury or infection. A transient increase in vascular permeability is a vital response to inflammation, allowing neutrophils to invade the inflamed tissue and clear the pathogen.

**Findings:**

The close interaction between neutrophils and the activated, inflamed endothelium, followed by leukocyte trafficking across the endothelial layer, can, however, also contribute to maladaptive vascular hyperpermeability. This increased permeability allows plasma proteins and fluid to escape into the surrounding tissue, leading to edema, a hallmark and frequent complication of many inflammatory disorders. Neutrophils also contribute to the resolution of inflammation by restricting further neutrophil recruitment through chemokine degradation, the formation of neutrophil extracellular traps (NETs), and by promoting their own apoptosis via the release of pro-apoptotic microparticles. Neutrophils, therefore, also contribute to the regulation of vascular permeability and to the restoration of tissue homeostasis in inflammatory conditions.

**Implications:**

This review summarizes the known mechanisms by which neutrophils regulate acute and chronic vascular permeability in autoimmune and non-autoimmune inflammatory diseases, and highlights the potential translational implications. Finally, we discuss overlapping and distinct mechanisms in neutrophil trafficking and vascular permeability.

## Introduction

Increased vascular leakage in inflammatory conditions largely depends on interactions between neutrophils and the vascular endothelium. Neutrophil-mediated vascular permeability occurs through physical neutrophil-endothelium interactions, mechanosignaling through adhesive molecules, “secretome” release, and neutrophil extracellular traps (NETs) formation. Neutrophil adhesion to endothelial cells (ECs) triggers their transendothelial migration (TEM) across the endothelial barrier into the underlying tissue, where they initiate pathogen clearance and inflammation resolution to restore tissue homeostasis. However, neutrophil-endothelial interactions can also lead to excessive activation of the endothelium and the subsequent recruitment of additional neutrophils, resulting in prolonged inflammatory responses and endothelial damage [1]. Therefore, the transient nature of inflammation-associated vascular permeability is essential to restore proper vascular physiology and prevent sustained hyperpermeability, which can lead to persistent tissue inflammation, hypotension, edema, and even organ dysfunction [2]. Prolonged vascular leakage indeed contributes to the pathology of several acute and chronic inflammatory diseases such as sepsis, coronavirus disease-19 (COVID-19), and thrombo-inflammatory and autoimmune diseases [3, 4]. Although the precise molecular mechanisms underlying neutrophil-EC interactions in physiological and pathological conditions remain incompletely understood, recent studies and advances in intravital imaging techniques have greatly expanded our understanding of the impact of neutrophil activation and extravasation on endothelial barrier integrity and its downstream consequences. Here, we review the mechanisms by which neutrophils influence vascular permeability and examine how uncontrolled neutrophil activation exacerbates and perpetuates inflammation through excessive damage to the endothelial barrier.

## Neutrophil-mediated signaling in vascular permeability

### Regulation of endothelial junctions

ECs line all blood vessels, regulate the delivery of oxygen and nutrients to surrounding tissues, and actively control vascular permeability by orchestrating immune-cell TEM through several molecules, proteins, and signaling pathways (Table 1). Although leukocytes can penetrate and wander through ECs, TEM primarily occurs via diapedesis, in which leukocytes cross the endothelial barrier at interendothelial junctions, which are typically composed of adhesive molecules implicated in vessel homeostasis [1, 5]. Under inflammatory conditions, these junctions become leaky due to complex cortical actin- and myosin-based cytoskeletal rearrangements at sites poised for increased vascular permeability [6]. The early responses to inflammation occur through a mechanosensory complex within adherent junctions (AJs) consisting of vascular endothelial (VE)-cadherin and its interaction partners, platelet endothelial cell adhesion molecule 1 (PECAM-1) and vascular endothelial growth factor receptor 2 (VEGFR2) [7], which control the structural integrity of the endothelium in various vascular beds [8]. Through its transmembrane domain, VE-cadherin mediates the interaction between PECAM-1 and VEGFR2, regulating the activity of the latter in response to PECAM-1-mediated mechanosignaling [7]. VE-cadherin-associated AJs are destabilized in response to extracellular stimuli permissive to neutrophil TEM, leading to increased barrier permeability [5]. This process is largely mediated by the phosphorylation of VE-cadherin at different residues by multiple proteins, including c-Src. The latter phosphorylates VE-cadherin at tyrosine residues Y658 and Y731, reducing its binding to β-catenin molecules, which anchor the resulting complex to the cytoskeleton [9, 10]. Under physiological conditions, phosphorylation of VE-cadherin is kept in check and counteracted by vascular endothelial protein tyrosine phosphatase (VE-PTP), which stabilizes VE-cadherin junctions by suppressing the rate of VE-cadherin internalization and therefore ensures the balance between junctional integrity and vascular permeability to prevent excessive vascular leakage [11]. Ligand-mediated activation of endothelial intracellular adhesion molecule 1 (ICAM-1) also plays a major role in neutrophil adhesion by inducing VE-cadherin phosphorylation [10]. Moreover, an elevation in calcium influx through PECAM-1-mediated activation of transient receptor potential channel 6 (TRPC6) is required for neutrophil diapedesis [12]. Other intracellular signaling pathways, such as Rho GTPases, including RhoA, Rac, and Cdc42, are implicated in neutrophil polarization and migration by rearrangement of the actin cytoskeleton [13], while PKA/PKG/AMPK-stimulated phosphorylations of the actin-regulating Ena/VASP proteins further fine-tune vascular permeability [14, 15]. However, pathological conditions associated with increased inflammation, neutrophil diapedesis, and vascular permeability often disrupt the regulatory mechanisms that govern controlled vascular permeability. For instance, increased vascular stiffness, a typical hallmark of inflammatory disease, is associated with failure of cell–cell junction formation mediated by VE-cadherin, which results in the rupture of barrier integrity and elevated vascular permeability [16].

**Table 1:** Endothelial molecules involved in vascular permeability and barrier integrity

Molecule	Mechanism	Function	Reference
(VE)-cadherin	Phosphorylating/dephosphorylating VE-cadherin at different residues and reducing VE-cadherin binding to β-catenin	Stabilize protein–protein adhesion and reduce barrier integrity	[9, 10]
ICAM-1	Phosphorylating VE-cadherin	Contribute to neutrophil adhesion	[10]
VE-PTP	Stabilizing VE-cadherin junctions by suppressing the rate of VE-cadherin internalization	Provide a balance between junctional integrity and vascular permeability	[11]
PECAM-1	Activating transient receptor potential channel 6 (TRPC6)	Elevation of calcium influx in neutrophil diapedesis	[12]
	Forming a complex with VE-cadherin	Trigger junctional rearrangement	[7]
Rho GTPases	Rearranging actin cytoskeleton	Neutrophil polarization and migration	[13]
	Phosphorylating actin-regulating Ena/VASP proteins	Control vascular permeability	[14,15]
L-selectin and PSGL-1	Binding to endothelial P- and E-selectin	Facilitate capturing and rolling of neutrophils on activated ECs	[47]

### Mechanistic roles of neutrophil-endothelial physical interactions in vascular permeability

The first essential step of neutrophil extravasation is their efficient adhesion to and interaction with the endothelium. Under physiological conditions, however, the quiescent endothelium functions as an anti-inflammatory surface that does not engage circulating neutrophils to preserve vascular homeostasis and barrier function. This is largely due to the endothelial glycocalyx (eGC), a negatively charged network of glycoproteins, proteoglycans, and glycosaminoglycans (GAGs) that repels the similarly negatively charged surfaces of neutrophils, thereby limiting their adhesion to the endothelium [17–19]. Anchored by core proteoglycans such as syndecan-1 and linked to the actin cytoskeleton, the eGC forms a physical and signaling barrier that restricts neutrophil access to endothelial adhesion molecules and modulates extracellular communication [17, 20–25]. Tissue damage or infection triggers endothelial cell activation, leading to eGC shedding, increased expression of pro-adhesive molecules, and consequent disruption of barrier integrity, which promotes endothelial dysfunction and microvascular permeability [26–30]. This compromised barrier facilitates coagulation activation, impairs mechanotransduction, and stimulates endothelial nitric oxide synthase and hence vasodilation, which significantly contribute to increased vascular permeability and enhanced access of neutrophils to the endothelial surface [21, 26, 31, 32]. As a result, adhesion molecules such as ICAM-1 and vascular adhesion molecule 1 (VCAM-1) are upregulated, promoting neutrophil attachment, accumulation, and subsequent extravasation as part of the host’s attempt to control infection [33, 34]. The GAG components of the eGC bind and immobilize key neutrophil-arrest and transmigration chemokines, such as interleukin(IL)-8, through electrostatic interactions between negatively charged GAG residues and positively charged arginine and lysine residues, thereby stabilizing chemokine gradients essential for directed neutrophil migration [35, 36]. Furthermore, syndecan cleaved by thrombin produces ectodomain fragments that promote AJs perturbation and stress fiber formation, facilitating increased paracellular permeability [37]. Together, these processes lead to the deceleration and eventual arrest of circulating neutrophils [38, 39], which firmly adhere to the endothelium before diapedesis and regulate vascular permeability through adhesion-dependent signals [40]. Several endothelial-derived surface mediators further fine-tune neutrophil rolling and adhesion; among the best studied are sphingosine-1-phosphate (S1P) and angiopoietin-2 (Angpt2). S1P, via its receptor S1PR, enhances leukocyte rolling and amplifies histamine- or epinephrine-induced rolling [41] by mobilizing endothelial P-selectin [42]. In contrast, Angpt2 does not affect rolling but promotes firm neutrophil adhesion to the endothelium, particularly in the presence of pro-inflammatory cytokines [43].

Neutrophils themselves also play an active and central role in many of the aforementioned events. Tissue damage or infection triggers resident, sentinel cells to secrete chemoattractants such as leukotriene B4 (LTB_4_) and leads to complement activation, which enables neutrophil recruitment [44]. Although neutrophil-derived LTB_4_ increases vascular permeability by promoting the release of heparin-binding protein from neutrophils [45], extravasation of neutrophils is independent of neutrophil-derived LTB_4_ [46]. Additionally, L-selectin and P-selectin glycoprotein ligand-1 (PSGL-1) on the neutrophil cell membrane bind to endothelial P- and E-selectin, respectively, facilitating their capture and rolling on activated ECs [47]. Activation of G protein-coupled receptors (GPCRs) on ECs triggers intracellular signaling cascades that lead to the expression and release of molecules such as VCAM-1, as well as cytokines like IL-8, a response that is crucial for neutrophil recruitment [48, 49]. Subsequent activation of neutrophil GPCRs initiates β2-integrin-mediated inside-out signaling involving Rap1, kindlin-3, and talin [50]. This ultimately results in firm adhesion of neutrophils to ECs via the interaction of primed β2-integrins such as adhesion molecules lymphocyte function-associated antigen-1 (LFA-1, or CD11a/CD18) and macrophage-1 antigen (Mac-1, or CD11b/CD18) expressed on neutrophils with their endothelial counterreceptors ICAM-1 and ICAM-2, respectively [51]. This adhesion is essential for the proteolytic activity of neutrophils, as it provides a closed space between neutrophils and the vascular wall to prevent access to inhibitory molecules such as alpha-1 antitrypsin, which would otherwise break down neutrophil-derived proteases [52, 53].

**Table 2:** Neutrophil secretome and mediators contribute to regulating vascular permeability

Molecule	Mechanism	Function	Reference
LTB_4_	Facilitating release of heparin-binding protein	Induce alteration in vascular permeability	[45]
NE	Degrading VE-cadherin components in an adhesion-dependent process	Disrupt EC junctions	[60–62]
	*Inducing IL-8 expression* through activation of TLR-4, which triggers *activation of MyD88 and NF*κ-B *signaling pathways*	*Increase vascular permeability* with attendant disruption of VE-cadherin junctions	[62]
	*Converting kininogens to bradykinin*	*Increase vascular permeability*	[63]
	Activating PAR2 signaling in ECs and reducing VE-cadherin expression	Induce vascular leakage by increasing contractile forces due to the formation of actin filaments	[60]
Cathepsin G	Inducing endothelial remodeling via the degradation of several matrix components and VE-cadherin	Contribute to neutrophil-mediated vascular leakage	[56, 67]
*MMPs*	Activating ERK1/2 and p38 pathways, with culminate in reduced VE-cadherin- and F-actin-containing junctions	*Perturb* EC integrity	[68]
MPO	Facilitating formation of oxidizing hypohalous acids	Influence vascular system and increase vascular permeability	[72]
	Disintegrating eGC architecture by an ionic interaction with heparan sulfate side chains	Implicate in neutrophil-mediated vascular dysfunction and initiate neutrophil adhesion	[73, 74]
ROS	Stimulating transient receptor potential (TRP) melastatin 2 (TRPM2) channels on ECs	Degrade adhesion molecules and disintegrating tissue and endothelial junctions	[79]
	Inducing TRPM2-mediated Ca^2+^ influx triggers c-Src signaling activation and VE-cadherin phosphorylation	Induce VE-cadherin internalization and opens the junctions that promote neutrophil extravasation	[79]
TNF-α, IL-1β, and CCL/CXCL proteins	Triggering MMP-9 release by neutrophils	Contribute to the endothelial disruption and increased vascular permeability	[69]
	Activating of kinase signaling such as PKC, p38, Src, and (PI3K)/Akt kinases	Contribute to vascular barrier dysfunction, vascular leakage, and tissue edema	[80, 85, 86]
	Activating TNF-α signaling	Increase vascular permeability	[80, 85, 86]
MRP-8/14	Inducing cytoskeleton rearrangements, cytosol tubulin polymerization, and loosening of intercellular endothelial junctions	Increase vascular permeability	[90]
	Enhancing expression of adhesion molecules such as ICAM-1 on ECs, and facilitating neutrophil-EC binding via Mac-1	Increase neutrophil migration	[88, 91]
	Stimulating production and secretion of proinflammatory cytokines such as IL-1β, IL-6, and IL-8	Induce persistent inflammatory environment and prolonged neutrophil migration and vascular leakage	[88, 92, 93]
NETs	Enriching mediators within the chromatin meshwork	Influence indirectly vascular permeability	[95, 96]
	Inducing conformational changes to the adherent junctions and actin cytoskeleton, H3Cit leads to the thinning of VE-cadherin and actin stress fiber formation	Compromise endothelial integrity	[95, 96]
NET-associated NE and MMP-9	Inducing VE-cadherin proteolysis	Impair endothelial integrity and increase endothelial permeability	[61, 97]
*NET-derived MMP-9*	*Diminishing endothelial PECAM-1 levels*	*Destabilize endothelial barrier*	[98]
NET-associated proteases	Secreting and shedding of chemoattractant agents	Promote neutrophil recruitment and amplify vascular leakage	[99, 100]
PAD4	Boosting NET formation	Induce vascular leakage	[104]

### Regulatory roles of the neutrophil secretome in vascular permeability

Aside from the physical interaction of neutrophils with the inflamed endothelium, vascular integrity under pathological conditions is also controlled by neutrophil-derived secreted mediators. Upon stimulation by inflammatory responses, neutrophils release bioactive molecules, including proteases, reactive oxygen species (ROS), NETs, cytokines, chemokines, chemoattractant factors, and other secreted molecules that promote vascular permeability (Table 2). These components are all directly or indirectly implicated in the cleavage of intercellular connections and extracellular matrix proteins that induce intracellular signaling pathways and cytoskeletal remodeling associated with vascular leakage [54, 55]. Additionally, they play a significant role in diapedesis via degrading VE-cadherin and the disassembly of the endothelial barrier, with detrimental effects on vascular permeability [56–59].

*Neutrophil serine proteases (NSPs),* such as neutrophil elastase (NE), cathepsin G, and the low-abundant elastase-related proteinase-4 (NSP4), are stored in the azurophilic granules of neutrophils and contribute to the non-oxidative pathway of intra- and extracellular pathogen removal. NE disrupts EC junctions by degradation of VE-cadherin complexes in an adhesion-dependent process, ultimately increasing vascular permeability and promoting leukocyte extravasation [60–62]. It also induces IL-8 expression through Toll-like receptor (TLR) 4 activation, which triggers myeloid differentiation primary response 88 (MyD88) and nuclear factor kappa B (NFκ-B) signaling to increase permeability by disrupting VE-cadherin junctions [62]. Activated neutrophil-derived NE further increases vascular permeability by converting kininogens to bradykinin, a potent endothelium-dependent vasodilator [63]. Local enrichment of the bradykinin-producing kallikrein-kinin system at cell surface proteoglycans regulates bradykinin formation and neutrophil diapedesis in inflammatory conditions [64–66]. NE also activates protease-activated receptor-2 (PAR2) signaling in ECs, leading to vascular leakage by reducing VE-cadherin expression and via contractile forces due to the formation of actin filaments [60]. Cathepsin G further contributes to neutrophil-mediated vascular leakage by the induction of endothelial remodeling through the degradation of several matrix components and VE-cadherin, which causes gap formation [56, 67]. 

*Matrix metalloproteinases (MMPs)* stored in gelatinase granules can perturb EC integrity, mostly through extracellular signal-regulated kinase-1/2 and p38 pathways, culminating in reduced VE-cadherin- and F-actin-containing junctions [68]. The inflammatory stimuli IL-1β and TNF-α also trigger MMP-9 release by neutrophils and thus can directly contribute to the endothelial disruption and increased vascular permeability [69]. Neutrophil *myeloperoxidase (MPO)* is another pro-inflammatory mediator that influences vascular permeability [70] and plays a significant role in neutrophil-mediated vascular dysfunction in inflammatory conditions [71], in part by disrupting endothelial integrity and inducing excessive vascular leakage by facilitating the formation of oxidizing hypohalous acids [72]. This enzyme is also implicated in neutrophil-dependent disintegration of the eGC architecture by an ionic interaction with heparan sulfate side chains that alter endothelial function and initiate neutrophil adhesion [73, 74]. Accordingly, suppressing MPO attenuates neutrophil degranulation and thereby inhibits neutrophil-mediated EC damage [75].

*ROS* is an essential part of the host’s immune defense against invading pathogens, but excess ROS can degrade adhesion molecules and disintegrate tissue and endothelial junctions [76, 77]. Activated neutrophils can act as a source for toxic ROS such as superoxide anion (O2^−^), hydrogen peroxide (H_2_O_2_), hydroxyl anions (OH^−^), hydroxyl radicals (OH•), and hypochlorous acid (HOCl) released into the circulation, which aggravate the inflammatory response and increase vascular permeability [78]. Activated neutrophils generate ROS via NADPH oxidases, which stimulate transient receptor potential melastatin 2 (TRPM2) channels on ECs [79]. TRPM2-mediated Ca^2+^ influx then triggers c-Src signaling activation and hence VE-cadherin phosphorylation, inducing VE-cadherin internalization and perturbed junctional integrity that promote neutrophil extravasation.

*Cytokines and chemokines* released by activated neutrophils, including TNF-α [80, 81], IL-1β [82, 83], and chemokine (C–C motif) ligand (CCL) / CXCL proteins [40, 84], all contribute to neutrophil-induced vascular barrier dysfunction, vascular leakage, and tissue edema. Activation of kinase signaling, including protein kinase C, p38, Src, and phosphatidylinositol 3-kinase (PI3K)/Akt kinases, as well as shedding of the eGC, are all implicated in increased EC permeability in response to TNF-α signaling [80, 85, 86]. Secreted TNF-α induces EC barrier disruption by phosphorylation of VE-cadherin, and disassembly of critical tight junction proteins through mechanisms that include protein kinase C (PKC) and p38 MAPK, leading to rearrangement of the actin cytoskeleton [85]. Additionally, neutrophils secrete IL-1β in a ROS-dependent manner, which elevates procoagulant activity and vascular permeability during inflammatory situations via thrombin signaling [82, 83]. Elevated neutrophil and endothelial expression of the chemokine macrophage inflammatory protein (MIP-1α) is associated with increased vascular permeability via activation of Rac/PAK kinases [40]. CXCL8, another chemokine released by neutrophils, specifically activates Rac1 via CXCR2 and PI3Kγ, which consequently phosphorylates VE-cadherin and results in vascular leakage [87].

*Neutrophil-derived secreted factors*, such as myeloid-related protein-8 and -14 (MRP-8/14; also known as S100A8 and S100A9), display elevated levels in patients with inflammatory diseases related to severe vascular leakage [88, 89]. Concordantly, MRP-8/14 heterodimers contribute to the increased vascular permeability and neutrophil migration by modulating cytoskeletal rearrangements, cytosol tubulin polymerization, and loosening of the intercellular endothelial junctions [90]. They also induce neutrophil infiltration by enhancing the expression of adhesion molecules such as ICAM-1 on ECs and facilitate neutrophil-EC binding via Mac-1 to increase neutrophil migration [88, 91]. As MRP-8/-14 belong to the group of danger-associated molecular patterns (DAMPs) and are endogenous ligands of TLR-4, these proteins further stimulate the production and secretion of various interleukins that result in a persistent inflammatory environment and prolonged neutrophil migration and vascular leakage [88, 92, 93]. Moreover, a recent study reported that sepsis-induced neutrophil rolling involves novel, blood-borne, neutrophil-derived elongated structures that induce the release of the MRP-8/-14 complex [94], potentially causing sustained inflammation and leakage.

*NETs—*DNA structures decorated with histone molecules, elastase, and MPO enzymes, among others—warrant special attention during the inflammatory response. While primarily known for their ability to trap pathogens and prevent infectious dissemination, NETs also function as scavengers that allow for the local enrichment of permeability mediators within the chromatin meshwork. They disrupt endothelial integrity by inducing conformational changes to AJs and actin cytoskeleton, where citrullinated histone H3 leads to disruption of VE-cadherins and defective actin stress fiber formation [95, 96]. NET-associated NE and MMP-9 activity further induce VE-cadherin proteolysis that leads to impaired endothelial integrity and enhanced permeability [61, 97], further compounded by NET-derived MMP-9-mediated destabilization of the endothelial barrier through its inhibitory effect on endothelial PECAM-1 levels [98]. The proteolytic activities of NET-associated proteases additionally promote neutrophil recruitment and amplify vascular leakage through the secretion and shedding of chemoattractant agents that negatively impact barrier integrity [99, 100]. Interestingly, ECs have an intrinsic albeit limited capacity to counteract excessive endothelial damage caused by VE-cadherin degradation, as they can internalize NET components only to a restricted extent [97]. Activated, inflamed ECs, however, promote excess NET formation and display enhanced susceptibility to NET-mediated barrier dysfunction [101], further tipping the scale towards more adverse disease outcomes. Neutrophil-derived proteases and histones embedded within NETs have been shown to directly injure ECs, disrupting tight junctions, and promoting leakage across the vascular wall by mechanisms detailed above. This heightened permeability has been demonstrated to exacerbate local inflammation and to create an environment that favours further neutrophil infiltration and amplifies tissue injury. Therefore, while NETs are a versatile and potent antimicrobial tool, their overproduction can shift from host defence to immunopathology, highlighting the delicate balance that neutrophils must maintain between effective pathogen control and preserving vascular integrity.

Excessive NET activity is regulated by circulating DNases, and a deficiency in extracellular DNase1 and DNase1-like 3 (DNase1L3), which break down NETs, dramatically increases NET-driven thromboinflammation in sepsis [102]. Vice versa, targeting NETs by hyperactive bioengineered DNases constitutes a promising therapeutic strategy for interference with NET-mediated leakage and possibly thrombosis [103]. Peptidyl arginine deiminase 4 (PAD4), an enzyme responsible for the citrullination of histone H3 (H3Cit), boosts NET formation and vascular leakage [104]. Consequently, PAD4 inhibition preserves endothelial integrity in a mouse model of arterial erosion [105], and *Pad4*-deficient mice display reduced organ dysfunction and improved survival after hemorrhagic shock and bacterial sepsis [106]. Similarly, degradation of NETs by DNase1 or the disruption of NET formation by inhibition of PAD4 reduces vascular permeability, elevates vascular repair, and improves functional recovery after stroke [107]. Likewise, decreasing NETs with DNase treatment diminishes vascular permeability and lung damage, and improves survival in mouse models of acute lung injury [108].

The clinical relevance of NET-mediated vascular permeability has been well-documented over the last few years. Indeed, vascular occlusions in patients with severe bacterial infections correlated with defective NET degradation and the formation of intravascular NET clots [102]. Similarly, elevated plasma NET biomarkers are associated with the severity and mortality of acute respiratory distress syndrome (ARDS), and lower plasma DNase1 levels are related to the development of sepsis-induced ARDS [108]. Although NET inhibition has gained significant traction as an alternative strategy to combat inflammation [109] and PAD4 inhibitors have shown clinical promise [110], eliminating the anti-inflammatory properties of NETs may have detrimental consequences in inflammatory disorders. This is perhaps best exemplified by the observations that *Pad4*^±^ mice display better outcomes in a mouse model of bacterial pneumonia than wild types, while *Pad4*^*−/−*^ mice perform worse [106]. Likewise, *Pad4* does not seem to play a role in the response to neutrophilia and experimental endotoxemia [111]. Hence, careful titration of the pro- and anti-inflammatory characteristics of NETs is essential in designing novel anti-inflammatory therapies that also attenuate pathological vascular leakage.

## Neutrophil-mediated pathogenesis of endothelial hyperpermeability

**Fig. 1:**
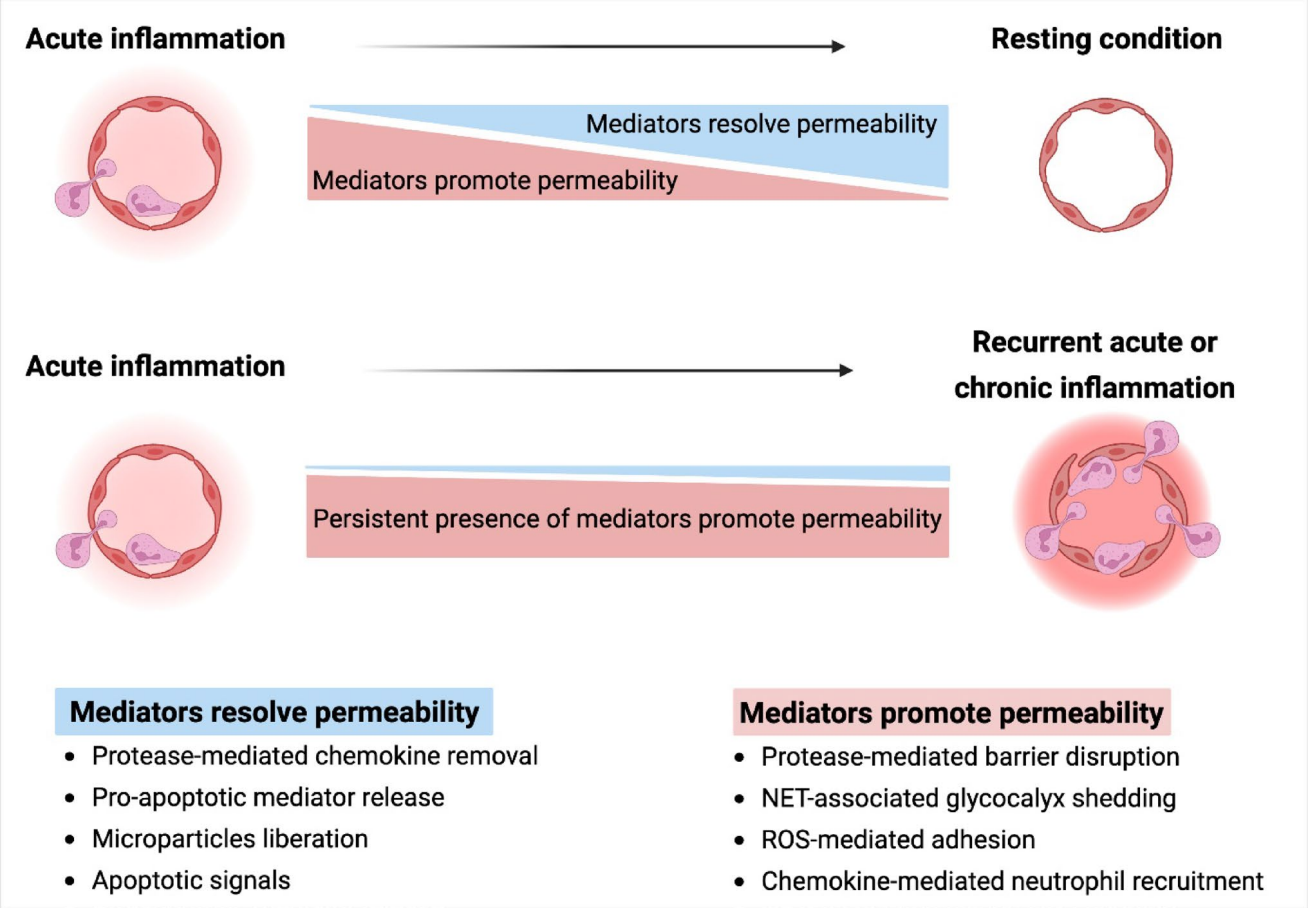
Mechanisms regulating vascular permeability in acute and chronic inflammation. In acute inflammation, neutrophil mediators promote transient vascular permeability and neutrophil infiltration and survival in the inflammatory milieu. During the evolution of inflammatory response, various neutrophil-mediated mechanisms regulate inflammation by reducing pro-inflammatory signals and providing pro-resolving mediators that establish tissue homeostasis and prevent excessive vascular leakage. Persistent production of inflammatory mediators in chronic inflammation leads to continuous vascular leakage. Created with BioRender

Vascular integrity is typically and efficiently restored following the elimination of pathogens and the resolution of inflammation [112], which is largely mediated by neutrophils through multiple complementary mechanisms, including the release of pro-resolving factors (Fig. 1). In acute inflammation, these pro-resolving mediators prevent excess vascular leak and re-establish tissue homeostasis [113, 114]. Neutrophils can also arrest further neutrophil influx via a NET-mediated chemokine degradation process, a process that not only results in the disruption of chemokine gradient-dependent neutrophil migration but also restores vascular quiescence [114, 115]. Moreover, neutrophils contain extracellular vesicles that trigger self-apoptosis and apoptotic clearance by releasing pro-apoptotic and anti-inflammatory microparticles, including Annexin 1 [114]. The latter initiates inflammation resolution by suppressing neutrophil adhesion to the endothelium, which improves endothelial function [116]. In concordance with these findings, increased vascular permeability has been observed in *Annexin A1*-deficient mice, which could be reversed by intravenous Annexin 1 injection [117].

Despite these mechanisms for preventing long-lasting vascular permeability, excess release of neutrophil components such as proteases and NETs and persistent inflammatory mediators leads to continuous vascular leakage and serious tissue dysfunction in recurrent acute inflammatory situations [118] (Fig. 1). Indeed, NET-induced shedding of the eGC can prime the endothelium for leukocyte adhesion and significantly contribute to an excessive inflammatory response [119]. Consistently, NETs are associated with increased vascular leakage in LPS-induced endotoxemia [104] and transfusion-related acute lung injury [120]. Additionally, ROS can extend neutrophil-EC adhesion and result in persistent vascular permeability and edema as ROS-mediated Ca^2+^ release stimulates the expression of P-selectin molecules on the EC surface, facilitating further neutrophil adhesion and recruitment [78].

Sustained neutrophil-mediated vascular leakage is not only observed in acute inflammatory situations but also in defined chronic conditions such as autoimmune diseases [2, 121] (Fig. 1). Neutrophils indeed trigger persistent EC dysfunction and vascular hyperpermeability in several chronic autoimmune diseases such as systemic lupus erythematosus (SLE), systemic sclerosis (SSc), and anti-neutrophil cytoplasmic antibody (ANCA)-associated vasculitis (AAV). Activated neutrophils infiltrate the endothelium and signal to monocytes and macrophages to release cytokines and chemokines that attract additional immune cells, thereby providing a pro-inflammatory milieu that culminates in edema formation and hemorrhaging [122]. Adhesion of primed neutrophils to the activated endothelium in SSc disease leads to increased ROS production, prolonging endothelial dysfunction and increasing susceptibility to fibrosis [123]. In autoimmune AAV, ANCA promotes the conversion of rolling neutrophils to a firm integrin-mediated adhesive neutrophil phenotype [124–128] and induces endothelial damage with microvascular hemorrhage and leukocyte infiltration as shown by intravital microscopy [129]. Moreover, ANCA promotes NET-formation [130], which in turn increases vascular leakage, causing subendothelial edema [124]. MPO-ANCA-activated NETs increase albumin permeability when incubated with ECs in vitro [131].

The latter is in line with the increasingly recognized role of NETs in vascular leakage in autoimmune diseases. Indeed, autoinflammatory responses are initiated in an antigen–antibody-specific manner, where circulating complexes of NET-autoantigens-autoantibodies modulate vascular inflammation and endothelial dysfunction [122, 132]. NET components, including proteases, further contribute to the pathogenesis of autoimmune diseases by their detrimental effect on ECs [132]. Neutrophil proteases and NETs also play a major role in SLE manifestation, where autoantibodies generated against the MMP-9 enzyme contribute to NET formation, and the increased MMP-9 levels on NETs activate endothelial MMP-2 to disrupt vascular integrity through EC damage and vascular dysfunction [133].

## Neutrophil-derived vascular permeability in autoimmune and non-autoimmune inflammatory diseases

### Autoimmune inflammatory diseases

#### ANCA-associated vasculitis (AAV)

**Fig. 2:**
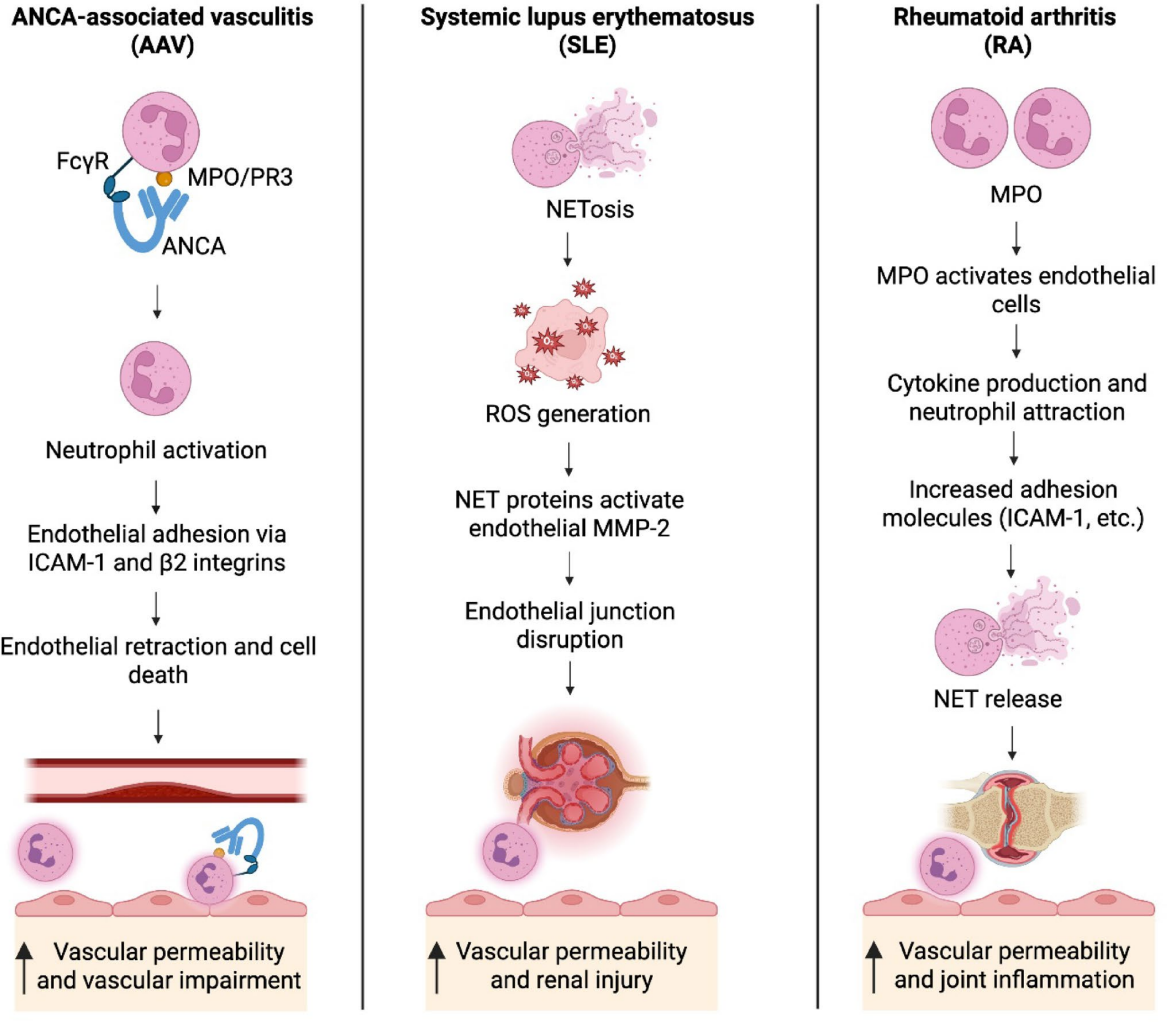
Neutrophil-mediated vascular permeability in autoimmune inflammatory diseases. The diagram illustrates distinct mechanisms by which neutrophils contribute to endothelial damage and vascular permeability in three autoimmune diseases: ANCA-associated vasculitis (AAV), systemic lupus erythematosus (SLE), and rheumatoid arthritis (RA). In AAV, ANCA target MPO or PR3, leading to neutrophil activation, endothelial adhesion via ICAM-1 and β2 integrins, and subsequent endothelial damage with retraction and EC death, resulting in vascular impairment. In SLE, NET formation (NETosis) induces ROS production and activation of endothelial MMP-2, leading to disruption of endothelial junctions and renal injury. In RA, MPO triggers endothelial cell activation and cytokine-mediated neutrophil recruitment. This promotes the expression of adhesion molecules (e.g., ICAM-1) and NET release, contributing to increased vascular permeability and joint inflammation. ANCA: anti-neutrophil cytoplasmic antibodies; MPO: myeloperoxidase; PR3: proteinase 3; ICAM-1: intercellular adhesion molecule 1; EC: endothelial cell; NET: neutrophil extracellular trap; ROS: reactive oxygen species; MMP-2: matrix metallopeptidase-2. Created with BioRender

AAV is characterized by systemic necrotizing inflammation of small blood vessels associated with highly specific proteinase 3 (PR3)- and MPO-ANCA [124, 127, 128, 134]. Neutrophils play a central role in the complex pathogenesis of AAV, as they are both targets of autoimmunity and effector cells that orchestrate endothelial and vascular injury [124, 128]. Interaction of ANCA with PR3 and MPO translocated from azurophilic granules to the cell surface, and Fcγ-receptor induces activation and degranulation of cytokine- and complement component C5a-primed neutrophils adhering to ECs. Proteolytic enzymes and oxygen radicals released by adherent ANCA-activated neutrophils cause EC death and fibrinoid necrosis of the vessel wall [124, 128, 135–137] (Fig. 2). ANCA-induced neutrophil activation in turn triggers activation of the alternative complement pathway, leading to the generation of C5a that binds to its receptor C5aR1 (CD88), mediating further neutrophil activation and thus, amplifying the inflammatory loop [127, 128, 138]. This pro-inflammatory effect, amplified by C5a, increases vascular permeability and the expression of EC adhesion molecules, acting as a chemoattractant to recruit further neutrophils [138]. Moreover, C5a activates C5a receptors on ECs and promotes P-selectin expression [139], cell retraction, and increased endothelial permeability [140]. Although ECs do not directly induce the complement system, their tight interaction with adherent neutrophils triggers surface complement and consequently results in complement-mediated EC dysfunction and increased vascular leakage [141]. Intravital imaging has shown the role of ANCA in converting rolling neutrophils to firm, stationary adhesive leukocytes [125, 126], which triggers an ICAM-1 and β2-integrins-mediated elevation of cytosolic free calcium, cytoskeletal rearrangements, and increased vascular permeability [40, 142]. Neutrophil adherence and TEM are further facilitated by EC detachment and vascular denudation, leading to increased microvascular permeability and exposure of the basement membrane and subendothelial matrix, resulting in the deposition of fibrin and platelets, as observed by electron microscopy in AAV glomerulonephritis [143]. Upon activation, these cells express P-selectin and display fibrinogen, which mediates neutrophil rolling, facilitates their arrest, and consequently supports their inflammatory recruitment [144]. As a result of vascular denudation, elevated levels of circulating ECs, a cellular marker for EC damage, have been observed in AAV patients, with their levels decreasing upon remission [145].

#### Systemic lupus erythematosus (SLE)

SLE is an autoimmune disease of unknown etiology with heterogeneous manifestations and significant dysregulation of innate and adaptive immunity, involving chronic systemic inflammation [146]. Neutrophils are central in SLE pathogenesis, exacerbating vascular damage and increasing the risk of developing atherosclerotic disease, particularly through the generation of ROS and the formation of NETs [147] (Fig. 2). NET proteins, such as MMP-9, induce endothelial dysfunction by increasing endothelial MMP-2 activity, leading to compromised vascular barriers and increased permeability [148]. The inflammatory milieu generated by activated neutrophils also contributes to nephritis, often observed in SLE patients, in which the renal vasculature is particularly affected [149–151]. This inflammatory environment can perpetuate a cycle of injury that contributes to the severe manifestations of SLE, including nephritis and other organ complications [149, 152]. The clinical importance of understanding neutrophil function in SLE is further emphasized by the potential for targeted therapeutic strategies to alleviate vascular complications. Suppressing NET formation and regulating neutrophil activity by using PAD4 inhibitors, NADPH oxidase inhibitors, and DNase therapy can potentially decrease the vascular damage that often accompanies SLE [153] and, therefore, could offer new opportunities in the development of treatment approaches to reduce disease severity and improve patient outcomes [154]. Uncovering neutrophil-related biomarkers could also increase disease monitoring and design treatment methods for individual patients [148, 151, 153].

#### Rheumatoid arthritis (RA)

RA is a systemic inflammatory autoimmune disease characterized by dysregulated immune cell activation, excessive inflammatory cell infiltration, and increased vascular permeability [155]. Neutrophils are the most frequent leukocytes in the synovial fluid and play a significant role in the pathogenesis of RA and the inflammation and joint damage associated with it [156]. Increased levels of several neutrophil enzymes, such as MPO, are present in RA plasma, synovial fluid, and tissue [157, 158]. Besides its microbicidal functions, MPO interacts with ECs, which increases endothelial permeability during inflammation [159] (Fig. 2). It also induces the production of inflammatory cytokines and attracts neutrophils to the site of inflammation to intensify inflammation [160]. Recent observations have additionally uncovered a significant role for NETs in RA, where they contribute to RA's inflammatory milieu and enhance vascular permeability [27]. Accordingly, elevated NET levels and their contents have been observed in RA patients [28].

### Non-autoimmune inflammatory diseases

#### Sepsis

**Fig. 3:**
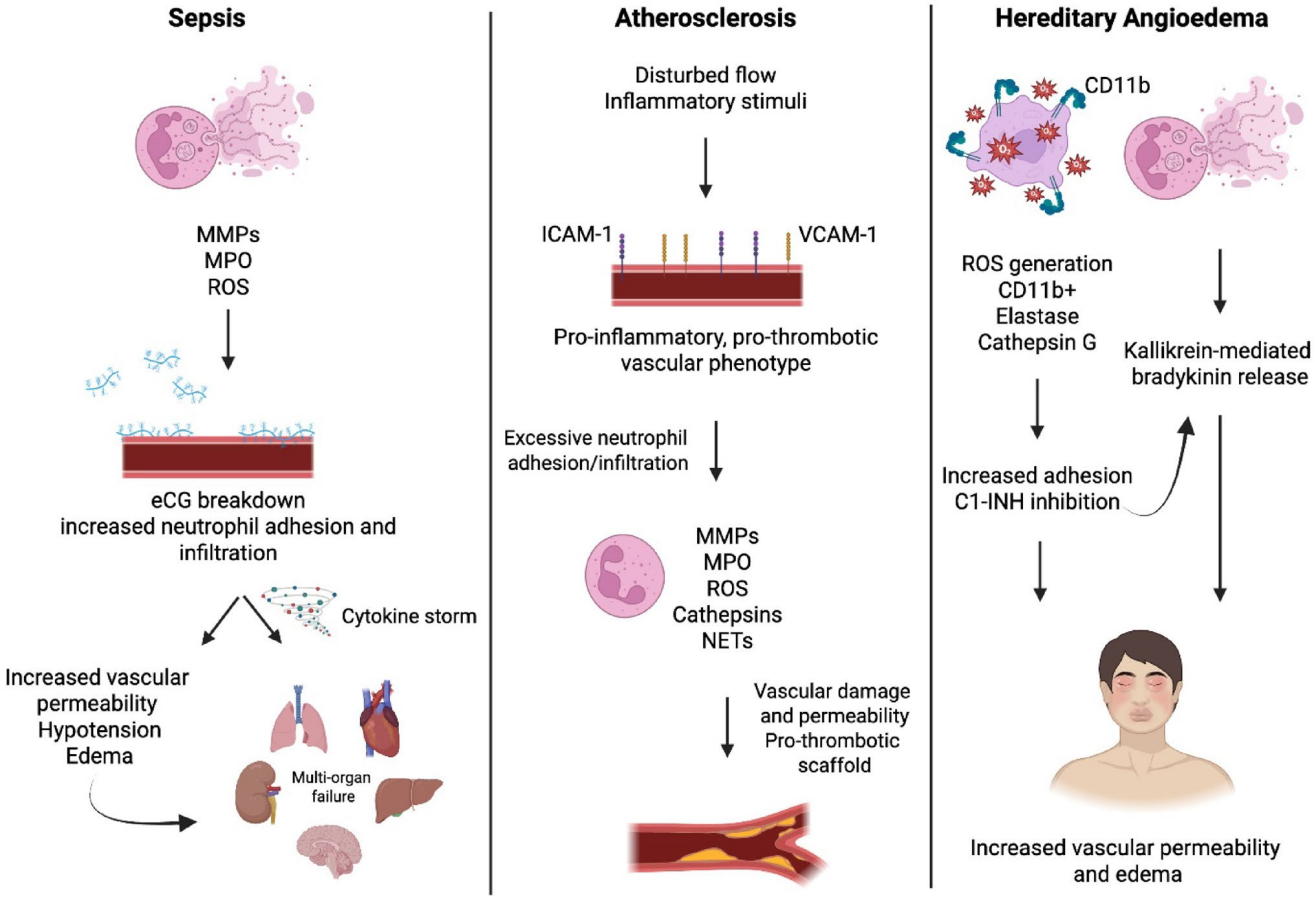
Neutrophil-mediated vascular permeability in non-autoimmune inflammatory diseases. This figure summarizes the contribution of neutrophils to vascular injury in sepsis, atherosclerosis, and hereditary angioedema. In sepsis, neutrophil-derived MMPs, MPO, and ROS degrade the eGC, increasing neutrophil adhesion and infiltration. This promotes a cytokine storm, vascular leakage, hypotension, edema, and multi-organ failure. In atherosclerosis, disturbed flow and inflammatory cues upregulate ICAM-1 and VCAM-1, promoting neutrophil adhesion. Activated neutrophils release MMPs, MPO, ROS, cathepsins, and NETs, leading to vascular damage, increased permeability, and a pro-thrombotic environment. In hereditary angioedema, neutrophil activation (via CD11b, elastase, and cathepsin G) impairs C1-INH, promoting kallikrein-mediated bradykinin release and increased vascular permeability, resulting in localized edema. MMPs: matrix metallopeptidases; MPO: myeloperoxidase; ROS: reactive oxygen species; eGC: endothelial glycocalyx; ICAM-1: intercellular adhesion molecule 1; VCAM-1: vascular cell adhesion molecule 1; NETs: neutrophil extracellular traps; C1-INH: C1 esterase inhibitor. Created with BioRender

Sepsis, a life-threatening organ dysfunction resulting from the dysregulated body's response to infection, is associated with perturbed vascular integrity and vascular hyperpermeability [161] (Fig. 3). This leads to the leakage of fluids and proteins into surrounding tissues, contributing to edema and hypotension and ultimately resulting in life‐threatening tissue injury and organ failure [162–165]. During disease progression, impaired ECs recruit numerous innate immune cells, including neutrophils, whose pro-inflammatory mediators trigger a cytokine storm and promote a pro‐inflammatory and pro‐coagulant vascular phenotype. Evidence indicates that pathogen-associated molecular patterns (PAMPs)-activated microvascular ECs induce endogenous stores of the heparan sulfate-specific glucuronidase, heparanase [166]. This enzyme cleaves heparan sulfate at the proteoglycan core of the eGC, thereby contributing to eGC degradation and extracellular matrix remodeling [167]. Additionally, bacterial products lead to elevated endothelial expression of various adhesion molecules through PAMPs and pro-inflammatory cytokines [168]. Together with the exposure of previously masked endothelial surface adhesion molecules due to the eGC loss, these triggers promote neutrophil adhesion to the endothelium, facilitate transmigration of further neutrophils into tissues, and contribute to endothelial barrier dysfunction and pulmonary endothelial hyperpermeability in sepsis and sepsis-induced acute lung injury [166]. Several studies have addressed a link between neutrophil ROS generation and the development of organ failure in sepsis [169, 170]. Exogenous ROS are released by recruited neutrophils when they receive a signal from physical, chemical, or biological stimuli. ROS can harm vascular ECs, resulting in elevated vascular permeability and tissue edema [170]. They can also compromise the vascular barrier and increase vascular permeability and platelet adhesion, leading to endothelial dysfunction [171]. Furthermore, NETs contain decondensed chromatin decorated with neutrophil components, which can directly influence the eGC as well as indirectly through the release of MMPs and MPO, contributing to eGC damage and degradation of junctional proteins such as VE-Cadherin, leading to increased vascular leakage [96, 165, 172].

#### Atherosclerosis and plaque development

Atherosclerosis is a chronic inflammatory disease of the arterial wall marked by vascular accumulation of fibrous elements and immune cells, such as neutrophils. Vascular dysfunction, which includes vascular permeability, has long been recognised as a hallmark of leakage in early atherogenesis [168]. Recent insights have revealed a pivotal role of neutrophils in modulating vascular barrier integrity during atherosclerotic disease progression (Fig. 3). In the initial stages of atherosclerosis, ECs respond to disturbed blood flow and inflammatory stimuli by upregulating the adhesion molecules P-selectin, E-selectin, ICAM-1, and VCAM-1, which facilitate the tethering, rolling, and firm adhesion of neutrophils to the endothelium. Chemokines such as CXC ligand 1 (CXCL1) and CXC ligand 8 (CXCL8; also known as IL-8) and lipid mediators such as LTB_4_ act as potent chemoattractants, promoting the transmigration of neutrophils into the subendothelial space. Following recruitment, neutrophils contribute to vascular permeability through several interrelated mechanisms, including the release of proteolytic enzymes such as NE, MMPs, and cathepsins. These enzymes have been shown to degrade components of the endothelial basement membrane and intercellular junctions, such as VE-cadherin, thus compromising EC integrity and increasing permeability [173]. Secondly, activated neutrophils generate excessive levels of ROS, which oxidatively modify junctional proteins and lipids, disrupt EC-cell contacts, and promote EC apoptosis, thereby further weakening the vascular barrier. Furthermore, it has been demonstrated that NETs can act as a scaffold for the activation of coagulation pathways, leading to the generation of thrombin and subsequent fibrin deposition. Thrombi disrupt perfusion, leading to vascular leakage, while thrombin-mediated signalling further compromises vascular barriers and increases permeability [174]. NETs not only act as pro-inflammatory and pro-thrombotic scaffolds in atherosclerosis, but also physically disrupt the endothelial lining via NET-associated proteases that cleave intercellular junctions and the cytotoxic effect of NET-associated histones on ECs [175]. Lastly, neutrophils amplify local inflammation through the secretion of cytokines and chemokines, such as IL-1β and S100A8/A9, which in turn recruit additional leukocytes to the affected area. This secondary wave of immune infiltration has been shown to cause further damage to the endothelial barrier, thereby creating a pro-permeability environment. Taken together, increased neutrophil-driven vascular permeability has significant consequences for atherogenesis.

#### Hereditary angioedema (HAE)

HAE is a rare, autosomal dominant disorder characterized by episodic skin swelling, gastrointestinal tract, and airway mucosa, primarily due to dysregulated bradykinin production. The majority of HAE cases are due to C1 esterase inhibitor (C1-INH) deficiency or dysfunction, leading to uncontrolled activation of the contact system and excessive bradykinin release [176]. Although traditionally viewed through the lens of complement and kallikrein-kinin system dysregulation, increasing evidence implicates neutrophils as important modulators of disease pathogenesis. In HAE, neutrophils are primed, evidenced by increased expression of adhesion molecules such as CD11b and an enhanced capacity to produce ROS [177, 178] (Fig. 3). NETs also trigger contact system activation by providing a scaffold for factor XII (FXII) autoactivation, and this activation initiates a proteolytic cascade that culminates in the generation of kallikrein and cleavage of high molecular weight kininogen (HMWK) to release bradykinin [179], leading to increased vascular permeability. This NET-mediated pathway represents an important amplification loop in HAE, particularly during acute attacks when neutrophil activation is enhanced. Neutrophil-derived proteases such as elastase and cathepsin G further degrade C1-INH to exacerbate its functional deficiency and perpetuate bradykinin overproduction [180]. The therapeutic implications of these findings are significant, as specific inhibitors targeting peptidylarginine deiminase 4 (PAD4), an enzyme critical for NETosis, and monoclonal antibodies against FXIIa (such as garadacimab) offer promising strategies to attenuate HAE attacks by intervening upstream in the neutrophil-contact system axis [180, 181]. In conclusion, neutrophils play a central role in the pathogenesis of HAE through mechanisms involving NET formation and contact system activation, resulting in enhanced vascular permeability and leakage.

## Neutrophil extravasation and vascular leakage: distinct but overlapping processes

Neutrophil accumulation and vascular leakage are the hallmarks of various critical acute and chronic illnesses such as acute lung injury, septic shock, acute respiratory distress syndrome, SLE, and RA [182, 183]. Although current evidence shows that neutrophil extravasation and vascular leakage are closely interconnected, the molecular mechanisms governing these processes are more complex than previously assumed and may even be partially uncoupled. Advanced intravital imaging and molecular cell biology techniques have indeed recently challenged the concept that vascular leakage is a natural consequence of neutrophil TEM, instead suggesting that these processes can occur independently [184–187]. Indeed, TEM and vascular leakage can occur at distinct sites within the vessel wall [188, 189], and some studies even report that increased vascular permeability can precede neutrophil extravasation [190]. Neutrophil adhesion via CD11/CD18 increases EC permeability and vascular leakage, irrespective of neutrophil TEM, further indicating that vascular permeability and diapedesis can be uncoupled [184, 191–193]. Moreover, neutrophil depletion does not influence vascular permeability in skin wounds [190]. Congruent with these data, several mechanisms that allow for efficient leukocyte TEM independent of increased vascular permeability have recently been identified. For instance, while dephosphorylation of tyrosine residue Tyr731 on VE-cadherin is involved in neutrophil TEM, leakage from vascular walls in response to inflammatory mediators requires phosphorylation of Tyr685 [194]. Reorganizations of small GTPase Rho A-mediated cytoskeleton have demonstrated another mechanism to prevent vascular leakage during neutrophil extravasation that regulates endothelial junctions [195]. Endothelial docking structures extend to dome-like shapes that further minimize vascular permeability in areas of active transcellular and paracellular TEM [196]. As recently shown by multi-photon intravital imaging, neutrophils penetrate a double-layered endothelial barrier mostly at specific sites of the vessel wall, so-called hotspots, which are regulated by distinguished roles of LFA-1 and Mac-1 molecules to help maintain barrier integrity and vascular homeostasis during inflammation [197].

**Fig. 4:**
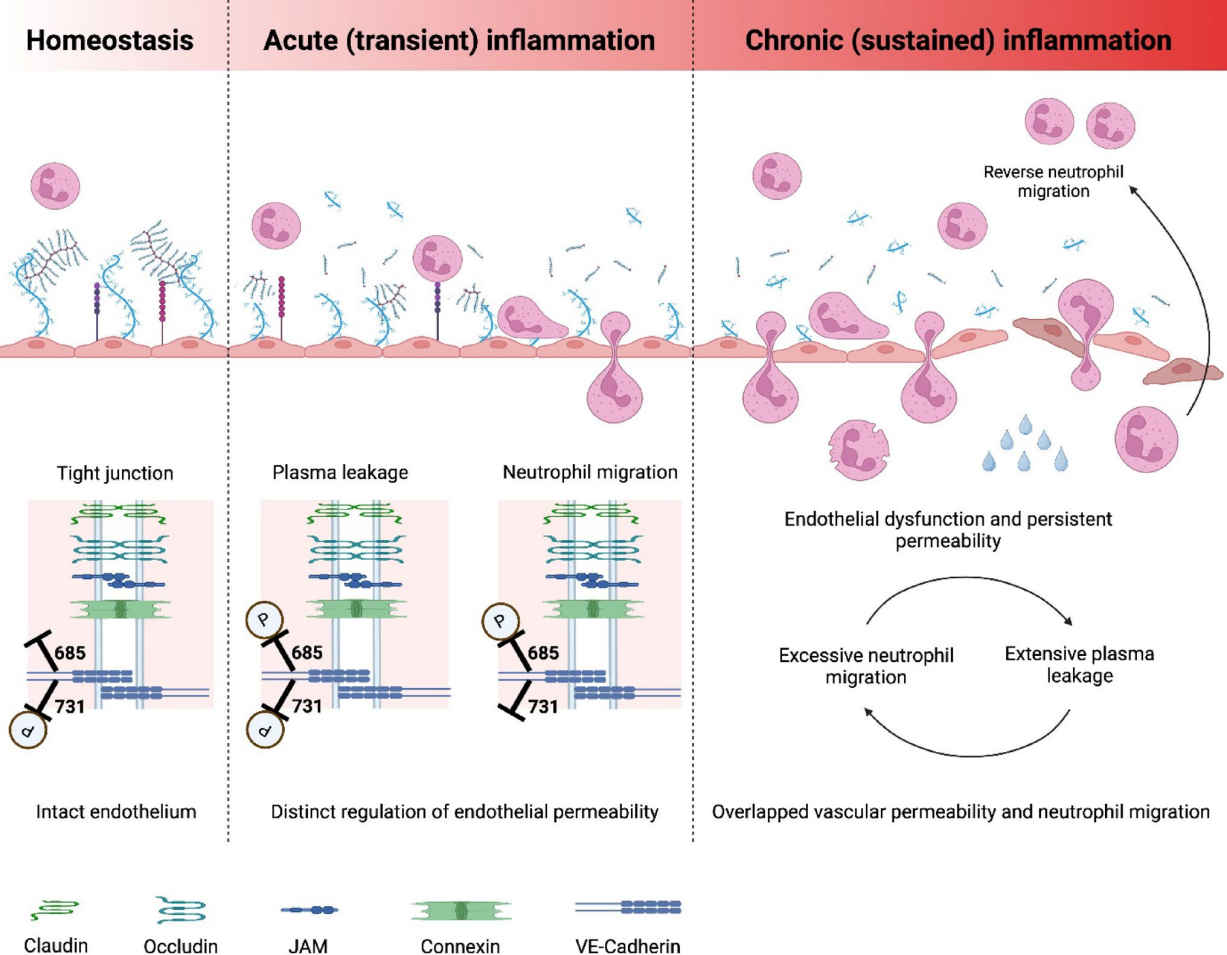
Overlapping but distinct regulation of vascular permeability and leukocyte extravasation. During acute or transient inflammation, damage to eGC, a delicate endothelium layer, increases vascular permeability, which can result in increased protein and water transit. Phosphorylation and dephosphorylation of specific tyrosines on VE-cadherin contribute to vascular permeability and neutrophil extravasation, indicating a distinct process in transient inflammation. An overshooting immune response induces tissue damage and organ dysfunction in sustained or chronic inflammation, leading to uncontrolled and overlapping vascular permeability and neutrophil extravasation. eGC: endothelial glycocalyx; VE: vascular endothelial. Created with BioRender

Although TEM and vascular leakage can be regulated through distinct mechanisms, they may overlap under severe inflammatory conditions (Fig. 4). Resolution of inflammation and restoration of barrier integrity are effective during the early stages of inflammation, but they fail to maintain endothelial function once inflammation becomes extensive and progressive, as large numbers of activated neutrophils accumulate and adhere to the vessel wall. Although neutrophils find specific spots for entry to the endothelium, these spots can be merged and become wider as the number of migration events increases [197]. Hence, examining both processes across early and advanced stages of inflammation, while accounting for the range of relevant inflammatory mediators, may provide a more comprehensive understanding of their regulation in human disease. Such studies could further unravel the common and divergent mechanisms governing vascular integrity and neutrophil migration and support the development of more targeted therapeutic approaches.

## Perspectives

Despite the well-documented interaction between neutrophils and ECs in inflammatory conditions, the precise mechanisms by which neutrophils induce vascular leakage and endothelial dysfunction remain incompletely understood. Although neutrophil TEM impacts vascular permeability, it is evident that activated neutrophils can also regulate barrier integrity by adhesion and secretome release, irrespective of diapedesis. The notion that neutrophil extravasation and vascular permeability do not necessarily coincide during inflammation could result in the development of novel therapies minimizing tissue edema and associated organ dysfunction while preserving immune response in inflammatory disorders. The complex nature of neutrophil-endothelial crosstalk is further determined by the precise stimuli that lead to inflammation, and these triggers may act in synergy or have opposite effects on endothelial integrity, vascular permeability, and, hence, disease outcomes in inflammatory disorders. A better understanding of context-dependent neutrophil-endothelial interactions, which integrates tissue- and disease-specific leukocyte and vascular biology, could therefore further refine current therapeutic tools and lead to more precise, highly efficacious anti-inflammatory therapies.

## Data Availability

No datasets were generated or analysed during the current study.

## Abbreviations

ARDS: Acute respiratory distress syndrome
AJs: Adherent junctions
AAV: ANCA-associated vasculitis
Angpt2: Angiopoietin 2
ANCA: Anti-neutrophil cytoplasmic antibody
C1-INH: C1 esterase inhibitor
H3Cit: Citrullination of histone H3
COVID-19: Coronavirus disease-19
DAMPs: Danger-associated molecular patterns
ECs: Endothelial cells
eGC: Endothelial glycocalyx
GAG: Glycosaminoglycans
GPCRs: G protein-coupled receptors
HAE: Hereditary angioedema
IL: Interleukin
ICAM-1: Intracellular adhesion molecule 1
LTB_4_: Leukotriene B4
NSP4: Low-abundant elastase-related proteinase-4
LFA-1: Lymphocyte function-associated antigen-1
MIP-1α: Macrophage inflammatory protein
Mac-1: Macrophage-1 antigen
MMPs: Matrix metalloproteinases
MRP: Myeloid-related protein
MyD88: Myeloid differentiation primary response 88
MPO: Myeloperoxidase
NFκB: Nuclear factor kappa B
NE: Neutrophil elastase
NETs: Neutrophil extracellular traps
NSPs: Neutrophil serine proteases
PAMPs: Pathogen-associated molecular patterns
PAD4: Peptidylarginine deiminase 4
PECAM-1: Platelet endothelial cell adhesion molecule 1
PKC: Protein kinase C
PR3: Proteinase 3
PAR2: Protease-activated receptor-2
PSGL-1: P-selectin glycoprotein ligand-1
S1P: Sphingosine-1-phosphate
S1PR: Sphingosine-1-phosphate receptor
SLE: Systemic lupus erythematosus
SSc: Systemic sclerosis
TLR: Toll-like receptor
TRPC6: Transient receptor potential channel 6
TRPM2: Transient receptor potential melastatin 2
TEM: Transendothelial migration
VE: Vascular endothelial
VEGFR2: Vascular endothelial growth factor receptor 2
VE-PTP: Vascular endothelial protein tyrosine phosphatase
